# High-Performance Solid Composite Polymer Electrolyte for all Solid-State Lithium Battery Through Facile Microstructure Regulation

**DOI:** 10.3389/fchem.2019.00388

**Published:** 2019-05-31

**Authors:** Jingjing Yang, Xun Wang, Gai Zhang, Aijie Ma, Weixing Chen, Le Shao, Chao Shen, Keyu Xie

**Affiliations:** ^1^School of Materials and Chemical Engineering, Xi'an Technological University, Xi'an, China; ^2^Shaanxi Coal Chemical Industry Technology Research Institute Co. Ltd., Xi'an, China; ^3^State Key Laboratory of Solidification Processing, School of Materials Science and Engineering, Center for Nano Energy Materials, Northwestern Polytechnical University and Shaanxi Joint Laboratory of Graphene (NPU), Xi'an, China

**Keywords:** all solid-state lithium battery, solid composite polymer electrolyte, microstructure, graphite-like carbonitride, electrochemical property

## Abstract

Solid composite polymer electrolytes are the optimal candidate for all solid-state lithium batteries, because of their enhanced ionic conductivities, long-life cycle ability and compatibility to lithium anode. Herein, we reported a kind of solid composite polymer electrolyte comprised of poly(ethylene oxide), graphitic-like carbon nitride and lithium perchlorate, which was prepared by a facile solution blending method. Microstructure of the solid composite polymer electrolyte was regulated by thermal annealing and interaction among components and was characterized by XRD, DSC, FTIR-ATR, and ROM. The obtained solid composite polymer electrolyte achieved an ionic conductivity as high as 1.76 × 10^−5^ S cm^−1^ at 25°C. And the electrochemical stable window and the lithium ion transference number, t_+_, were also obviously enhanced. LiFePO_4_/Li solid-state batteries with the annealed PEO-LiClO_4_-g-C_3_N_4_ solid polymer electrolyte presented a high initial discharge capacity of 161.2 mAh g^−1^ and superior cycle stability with a capacity retention ratio of 81% after 200 cycles at 1C at 80°C. The above results indicates that the thermal annealing treatment and g-C_3_N_4_ as a novel structure modifier is crucial for obtaining the high-performance solid composite polymer electrolytes used in the all solid-state lithium battery.

## Introduction

During recent years, high-energy-density storage batteries are urgently needed to satisfy the increasing demand in electric vehicles, consumer electronics and grid energy storage (Manthiram et al., 2017; Fan et al., 2018). Lithium metal secondary batteries have been considered as the potential candidate for high-energy-density storage batteries, since lithium metal anode possessed the highest theoretical specific capacity (3,862 mAh g^−1^) and the lowest reduction potential (−3.04V VS. standard hydrogen electrode) (Shen et al., 2018; Wu et al., 2018). Moreover, lithium metal used as the anode can even act as the lithium source in the battery with non-lithiated materials such as sulfur or oxygen as the cathode to achieve higher storage capacity compared to the current commercial lithium-ion batteries (Pan et al., 2019). However, practical and large-scale development of lithium metal batteries with liquid electrolytes is limited by safety concerns and the rapid capacity attenuation and short cycling capability (Fan et al., 2018). During the charge-discharge process of lithium metal secondary batteries with organic liquid electrolytes, the propensity of faster lithium dendrites formation was resulted from the plating and stripping of Li ions on the anode, which would result in the capacity attenuation and poor cycling capability and short-circuit of cells.

In order to further develop the practical use of lithium metal as anode, scientists made great efforts to solve the lithium dendrites problems and achieved much progress on suppressing dendrite formation and growth (Li D. et al., 2018; Nie et al., 2018; Shen et al., 2018; Wu et al., 2018; Pan et al., 2019). Strategies such as electrolyte modification, multifunctional barriers, composite metallic lithium electrode, and 3D current collectors were proposed to suppress the formation of Lithium dendrites (Wu et al., 2018; Xiao et al., 2018). Replacement of current organic liquid electrolytes with the solid-state electrolytes is considered as the most promising way to realize excellent performance of lithium metal secondary batteries (Goodenough, 2018; Nie et al., 2018). Because the organic liquid electrolyte is flammable, toxic and environmental contamination, solid-state electrolytes are therefore of crucial importance because of their excellent safety and mechanical ability for dendritic growth inhibition (Ban et al., 2018). And all solid-state lithium metal batteries are widely regarded as promising candidates for next generation of energy storage devices with improved energy density and superior safety performances.

Solid-state electrolytes are generally divided into inorganic superionic conductors (or the solid ceramic electrolytes) and solid polymer electrolytes. Compared with the inorganic superionic conductors, solid polymer electrolytes, benefiting from shape versatility, flexibility, light weight and low processing costs, are being investigated as promising candidates to replace currently available organic liquid electrolytes in lithium metal batteries (Bae et al., 2018b), (Zhang X. et al., 2018). However, due to the inherent low ionic conductivity at RT and poor mechanical property of solid polymer electrolytes, polymer-based solid composite electrolytes taking advantage of the merits of both inorganic and polymeric materials have been attracting more and more attention in all solid-state lithium metal battery applications (Tan et al., 2018). And Poly(ethylene oxide) (PEO) based solid composite electrolytes were widely considered as promising candidates solid electrolytes for high energy density lithium metal batteries (Xue et al., 2015; Tan et al., 2018). Numerous fillers were added into the PEO-based composite solid electrolyte to improve the ionic conductivity and the interfacial properties in contact with the electrodes (Tan et al., 2018). And it was the simplest method to achieve the composite polymer electrolyte, whose physical properties can be easily controlled by compositional change (Tan et al., 2018; Xiao et al., 2018; Zhang J. et al., 2018). However, the particle agglomeration especially at high concentration of fillers would inhibit the further improvement of properties of the solid polymer electrolyte. New kind of effective filler or percolated nanofiller structure needs to be developed by researchers.

Graphitic-like carbon nitride (g-C_3_N_4_) is a stacked 2D structure, which was mainly composed of carbon, nitrogen and a few residual –NH_2_ or –NH groups, and was metal-free and lightweight (Wang et al., 2012; Shi et al., 2014). And its intrinsic polarity and semiconductor property may be helpful to modulate the spatial and interface distribution of ionic charge carriers (Hu et al., 2017). These properties make g-C_3_N_4_ a potential candidate as polymer electrolyte filler for all-in-solid lithium batteries. Shi et al. proved that the introduction of 6.0 wt% g-C_3_N_4_ nanosheets in the sodium alginate (SA) nanocomposite films could greatly enhance the thermal stability and mechanical properties of SA biopolymer electrolyte nanocomposite films (Shi et al., 2014). Hu et al. reported about a kind of composite electrolyte with bis(triffuoromethanesulfonimide) lithium salt (LiTFSI), di(ethylene glycol) dimethyl ether (DGM) and g-C_3_N_4_, which was used to suppress lithium dendrite growth during cycling and improve the long-term cycling property of the composite electrolyte (Hu et al., 2017). But quantitative and even qualitative descriptions of the interactions between separate components of the state electrolyte system are still lacking. Understanding of the different interactions in the system and its influence on the microstructure and final electrochemical properties of the solid composite polymer electrolyte is crucial for achieving high performance solid polymer electrolyte.

In this article, we present for the very first time, to the best of our knowledge, the addition of g-C_3_N_4_ as fillers in PEO and its influence on the lithium ionic dissociation in lithium perchlorate (LiClO_4_) and transport and the crystallization of PEO in solid composite polymer electrolytes. Thermal annealing treatment and the interactions regulation among components by addition of g-C_3_N_4_ were introduced to tailor the microstructure of the solid composite polymer electrolyte. And through controlling the microstructure of solid composite polymer electrolytes, excellent improvement of the desired physical and chemical properties and electrochemical properties was achieved. When the weight percent of g-C_3_N_4_ was 10%, the annealed solid composite polymer electrolyte gave the best performance in the all solid-state lithium battery with lithium metal as the anode and LiFePO_4_ as the cathode.

## Experimental Section

### Materials

Poly(ethylene oxide) (Mw = 6 × 10^5^, Alfa Aesar), lithium perchlorate (LiClO_4_, Aladdin, battery grade), poly(vinylidene fluoride) (PVDF, Solef^®^ 5130, Solvay), and LiFePO_4_ (DY-3, Shenzhen Dynanonic Co., Ltd) were commercially obtained and were dried before usage. Acetonitrile (ACN, Aladdin, AR), N-methly-2-pyrrolidone (NMP, AR), and urea (Aladdin, AR) were used as obtained. The Li metal used in our experiments was commercially obtained with a diameter of 15.8 and 0.6 mm of thickness.

### Preparation of g-C_3_N_4_

The g-C_3_*N*_4_ filler was synthesized by thermal polymerization as we reported in the previous article (Wang et al., 2019). Twenty gram of urea was put into a crucible with a cover in the muffle furnace. And then the crucible was heated to 580°C to stay for 4 h, with a heating rate of 3°C/min. And the powder product was in light yellow color. The detailed characterization of pure g-C_3_N_4_ was displayed in Figures S1, S3.

### Solid Composite Polymer Electrolyte Films Preparation

Firstly, the prepared g-C_3_N_4_ filler was ultrasonic dispersed in ACN. Then PEO and LiClO_4_ (molar ratio of ethylene oxide (EO) to LiClO_4_ is 15:1) were added into the solution under stirring, in order to obtain uniform mixture solution. The weight percentage of g-C_3_N_4_ compared to PEO was controlled as 0, 5, 10, 15, and 20%, respectively. The mixture solution was casted into a home-made Teflon mold and dried in the vacuum oven at 40°C for 48 h. And the above mentioned state polymer electrolytes films were firstly annealed at 120°C for 2 h and then quenched to RT before use. In contrast, the state polymer electrolyte films were also prepared without thermal annealing process. The thickness of obtained solid state polymer electrolyte films was regulated to about 100 μm. And the state polymer electrolytes films were cut into rounds with diameter of 19 mm before loaded into the glovebox.

### Characterization

Bruker Vertex 70 FTIR spectrometer with Access ATR^TM^ attachment was used to characterize the composition of samples. The melting temperature of PEO in samples was collected by a differential scanning calorimeter (DSC, Mettler DSC823e) under nitrogen atmosphere. Samples with a mass of 5~10 mg were sealed into aluminum crucible, and then heated at a rate of 10°C/min. X-ray powder diffraction (XRD) patterns were collected by a XRD-6000 (Shimazu, Japan) X-Ray diffractometer equipped with Cu K_α_ radiation in the range of 5~50° at a scanning rate of 4°/min. The reflection optical microscopy (ROM) observations were performed with a Leica DM2500P optical microscope equipped with a C-5050ZOOM camera.

The ionic conductivity of SPEs was measured by electrochemical impedance spectroscopy (EIS) by using a Princeton PARSTAT 4000A (AMETEK) electrochemical workstation over frequency range from 1 to 10^5^ Hz with an applied voltage of 10 mV. The measured SPEs were sandwiched between two stainless steel electrodes, which were assembled into CR2025 coin cells. And the temperature was controlled by an oven in the range of 25~120°C. The electrochemical stability window was determined by linear sweep voltammetry (LSV) by a Princeton PARSTAT 4000A (AMETEK) electrochemical workstation. The cell was assembled by sandwiching SPEs between a stainless steel and lithium metal (SS/SPE/Li) as the counter and reference electrode, respectively. And the measurement was cycled from 2.0 to 6.0 V (VS. Li/Li^+^) at a scan rate of 10 mV/S. The lithium transference number (t_+_) was tested in a symmetric cell (Li/SPE/Li) by chronoamperometry combined with electrochemical impedance spectroscopy according to the method provided by Bruce et al. (Evans et al., 1987). In order to characterize the battery performances, the prepared state polymer electrolytes were fabricated as CR2025 coin-type cells with lithium metal anode and LiFePO_4_ cathode. And LiFePO_4_ was blended with carbon black (Super-P) and poly(vinylidene fluoride) at a weight ratio of 8:1:1 with NMP as the solvent. The weight of cathode used in the test was controlled to about 2.40 mg. As a result, the weight of active material was about 1.92 mg. The battery performances of coin cells were examined at 80°C using a Battery Testing System (NEWARE CT-4008, Shenzhen, China), including cycle property and C-rate capability (0.1, 0.2, 0.5, 1, 2, and 5C). The cut-off voltages were chosen as 4.2 V (charge) and 2.5 V (discharge) during the charge-discharge cycle performance.

## Results and Discussion

### Microstructure Regulation by Thermal Annealing and Interaction Among Components in Solid Composite Polymer Electrolytes

**Figure 1:**
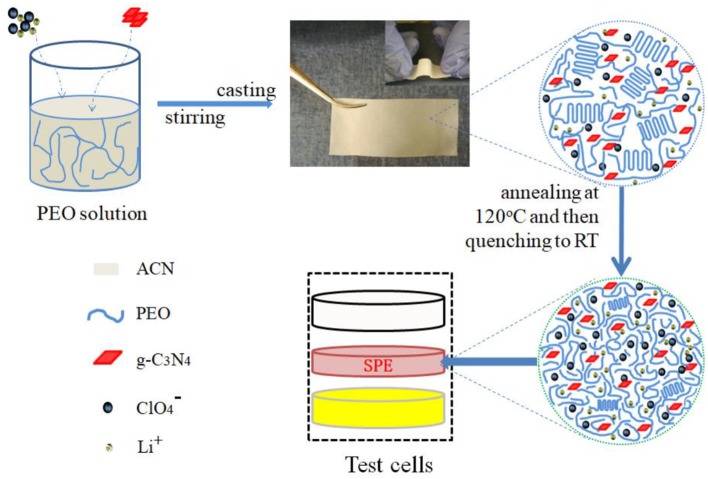
The flowchart of the preparation of the PEO-LiClO_4_-g-C_3_N_4_ solid composite polymer electrolyte film. Also the illustration of the microstructure was shown in the enlarged diagrams and the package method of test cells was shown.

In order to avoid the effect of thermal history on the crystallization of PEO during the solid composite polymer electrolyte preparation process to an extreme, 120°C annealing and then quenching method was introduced, as shown in Figure 1. And we have reported on this preparation method in detail in a previous work (Wang et al., 2019). During the solid polymer electrolyte preparation process, samples were firstly dried at 40°C for 48 h, which would provide an isothermal crystallization condition for PEO. As a result, the crystallinity of PEO in the samples was high. However, when the sample was quenching from the amorphous state, the non-isothermal crystallization might happened, which gave a lower crystallinity of PEO in the sample. And this deduction was further proved by the following XRD and DSC results.

**Figure 2:**
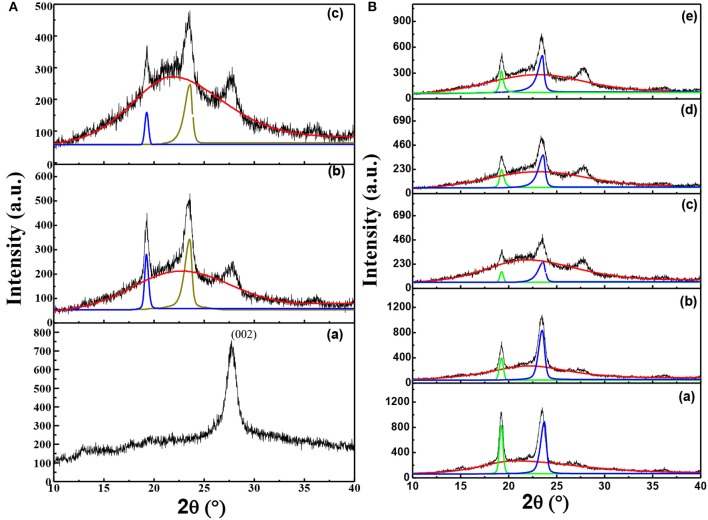
**(A)** XRD patterns of g-C_3_N_4_(a), solid composite polymer electrolytes with 10% g-C_3_N_4_ prepared without thermal annealing at 120°C (b) and with thermal annealing (c); **(B)** XRD patterns of solid composite polymer electrolytes with different weight percent of g-C_3_N_4_ [(a) 0%; (b) 5%; (c) 10%; (d) 15%, and (e) 20%] at the same [EO]: [Li_+_] ratio of 15:1.

In order to clarify the influence of thermal annealing and the interaction among components of the solid composite polymer electrolyte on the microstructure of solid composite polymer electrolytes, XRD was used to characterize the crystallization of samples as shown in Figure 2. As shown in Figure 2Aa, pure g-C_3_N_4_ gave a typical diffraction peak at 27.5°, which was the typical peak of (002) crystal plane of g-C_3_N_4_. Moreover, the interlamellar spacing of g-C_3_N_4_ was calculated as about 0.32 nm by Bragg equation. On the other hand, XRD patterns of the solid composite polymer electrolyte samples shown in Figures 2Ab,c displayed three typical peaks at 19.3°, 23.4° and 27.5°, respectively. The two characteristic peaks at 19.3° and 23.4° were corresponded to (120) and (132)/(032)/(212)/(112) crystal planes of PEO, respectively (Wang et al., 2019). It was obvious that the intensity of typical diffraction peak of PEO at 19.3° and 23.4° greatly decreased after the sample annealing at 120°C and then quenching to RT, as shown in Figures 2Ab,c.

Furthermore, the diffraction peaks of crystalline phase and amorphous phase in XRD patterns were fitted by the Jade 6.0 software. And the crystallization degree of PEO in samples was calculated by divided the summation of peak areas of crystallization phase and amorphous phase by the summation of peak areas of crystalline phase. Therefore, the crystallization degree of PEO was decreased from about 15.49% to about 8%, when the solid composite polymer electrolyte was thermal treated. This result demonstrated that the thermal annealing process could greatly suppress the crystallization of PEO.

As shown in Figure 2B, the intensity of diffraction peak observed at 27.5° increased with the content of g-C_3_N_4_ increasing. Figures 2Ba–e showed that intensity of typical peaks of PEO crystals was firstly decreased and then increased due to the addition of g-C_3_N_4_, with a minimum value appeared in the solid composite polymer electrolyte with 10% g-C_3_N_4_. Moreover, when the content of g-C_3_N_4_ was chosen as 0, 5, 10, 15, and 20%, the crystallization degree of PEO was calculated as about 28, 25, 8, 19, and 20%, respectively. It was obvious that the introduction of g-C_3_N_4_ could significantly decrease the crystallinity of PEO. But the crystallization degree of PEO was firstly decreased and then increased as the content of g-C_3_N_4_ increased, with a minimum value in the solid composite polymer electrolyte with 10% g-C_3_N_4_. In the state polymer electrolyte, the porous microstructure of g-C_3_N_4_ sheets (as shown in Figure S1) might serve as the hard limitation and inhibit the ordered chain arrangement of PEO, while on the other hand also serve as the nucleation sites for PEO chains. As a result, the heterogeneous nucleation could be enhanced, when the content of g-C_3_N_4_ sheets was over 10%, which would improve the crystallization of PEO. Furthermore, the g-C_3_N_4_ nanosheets might change the priority growth of PEO crystal planes, as shown by the relative intensity of fitting peaks at 19.3° and 23.4° in Figures 2Ba,e. It implied that the interaction between g-C_3_N_4_ and PEO could greatly influence the crystallization preference of crystal planes in PEO.

**Figure 3:**
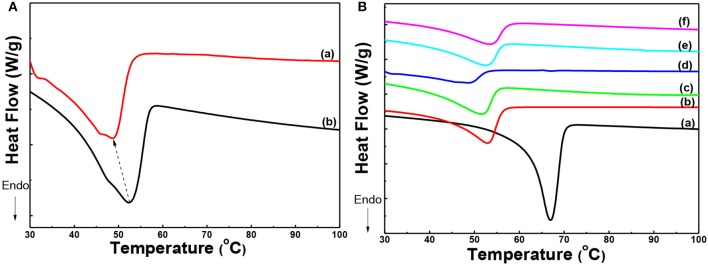
**(A)** DSC curves of solid composite polymer electrolytes prepared with thermal annealing at 120°C (a) and without thermal annealing (b); **(B)** DSC curves of PEO (a) and solid composite polymer electrolytes with different weight percent of g-C_3_N_4_ annealed at 120°C [(b) 0%; (c) 5%; (d) 10%; (e) 15%, and (f) 20%] at the same [EO]: [Li^+^] ratio of 15:1.

The effect of thermal annealing and the interaction among components of the solid composite polymer electrolyte on the crystallization of PEO was further proved by DSC. As shown in Figures 3Aa,b the melting temperature of PEO was decreased from 52.4 to 48.7°C, when the state polymer electrolyte was annealed at 120°C and then quenched to RT. Also shoulder peak appeared at 46.0°C in Figure 3Aa. It meant that PEO crystals in the sample undergone thermal annealing formed lamellae with thinner thickness and the thickness of lamellae gave a wider distribution. It implied that the thermal annealing process could regulate the crystallization of PEO.

Furthermore, the melting behavior of PEO in PEO homopolymer, PEO-LiClO_4_ electrolyte and the solid composite polymer electrolytes with different content of g-C_3_N_4_ (5, 10, 15, and 20%), which were treated by the thermal annealing process, was displayed in Figure 3B. Due to the interaction between PEO chains and LiClO_4_, the melting temperature of PEO crystals decreased from 66.7 to 53.3°C, as shown in Figures 3Ba,b. Moreover, the addition of g-C_3_N_4_ further restrained the crystallization of PEO, as shown in Figures 3Bc–f. The melting temperature of PEO crystals in the solid composite polymer electrolyte was 51.5, 48.7, 52.3, and 53.1°C, respectively, with the content of g-C_3_N_4_ increasing from 5 to 20%. The melting temperature of PEO firstly decreased and then increased, with a minimum value of 48.7°C in the sample with 10% g-C_3_N_4_. Also the crystallinity of PEO was calculated by dividing the melting enthalpy obtained from DSC curves in Figure 3B by the equilibrium enthalpy of PEO fusion (197 J/g). And the crystallinity of PEO in different samples shown in Figure 3B was about 54, 25, 22, 7, 18, and 20%, respectively. With the addition of LiClO_4_, the coordination effect of EO unit and Li^+^ would decrease the crystallization of PEO. Moreover, the introduction of g-C_3_N_4_ further decreased the crystallization of PEO. The residual –NH_2_ or –NH groups presenting in g-C_3_N_4_ can act as active sites for hydrogen bonding with ether oxygen in PEO. And the hydrogen bonds formation restrained the chain folding of PEO, and then reduced the crystallinity of PEO in the solid composite polymer electrolytes. On the other hand, the existence of g-C_3_N_4_ also served as the heterogeneous nucleation sites for PEO crystallization. As a result, the crystallinity degree of PEO firstly decreased and then increased slightly in the solid composite electrolytes, with 10% as the optimum content. This result was in good accordance with the foregoing XRD results.

**Figure 4:**
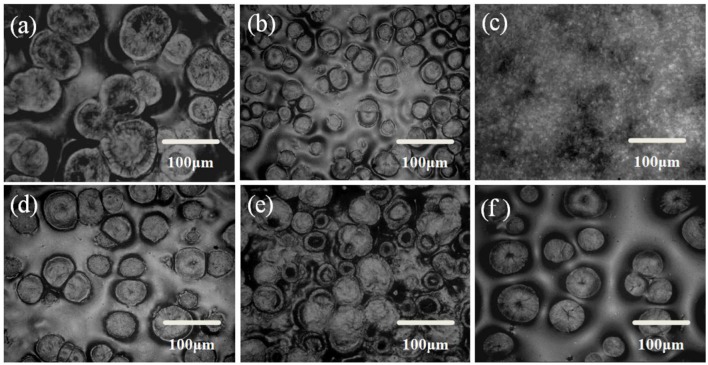
ROM images of solid composite polymer electrolytes prepared with thermal annealing at 120°C [**(a)** PEO-LiClO_4_; **(b)** PEO-LiClO_4_-5% g-C_3_N_4_; **(c)** PEO-LiClO_4_-10% g-C_3_N_4_; **(d)** PEO-LiClO_4_-15% g-C_3_N_4_; **(e)** PEO-LiClO_4_-20% g-C_3_N_4_] and without thermal annealing [**(f)** PEO-LiClO_4_-10% g-C_3_N_4_].

The influence of thermal annealing and the interaction among components of the solid composite polymer electrolyte on the microstructure morphology of solid composite polymer electrolyte was observed by ROM. Perfect spherulites with diameter of about 100 μm were formed in the solid PEO-LiClO_4_ polymer electrolyte, as observed in Figure 4a. Figures 4b–e showed that the morphology perfection and the crystal's size and number of PEO obviously changed with the content of g-C_3_N_4_. PEO spherulites were firstly disappeared and then re-formed with the weight percent of g-C_3_N_4_ increasing, with 10% as a critical content. It was ascribed to the hydrogen bonding interaction between g-C_3_N_4_ and PEO that could inhibit the crystallization of PEO, while g-C_3_N_4_ could also serve as the heterogeneous nucleation sites for PEO. Therefore, when the content of g-C_3_N_4_ was over 10%, more and more spherulites were formed in the sample. It implied that the interaction between PEO and g-C_3_N_4_ could greatly affect the morphology of the solid composite polymer electrolyte.

Moreover, compared Figure 4c with Figure 4f, the thermal annealing treatment might greatly change the morphology of PEO crystals from spherulites to lamellae stack. It demonstrated that the thermal treatment also influenced the morphology of the solid composite polymer electrolyte. The morphology of the solid composite polymer electrolyte was tailored by the thermal treatment and the interactions among components in the sample. And the morphology would play an important role in the compatibility between solid electrolytes and electrodes. Uniformly distributed PEO crystals might control the nucleation of lithium, which would help to inhibit the lithium dendrite formation.

### Effects of Interaction Among Components in Solid Composite Polymer Electrolytes on the Lithium Salt Dissociation

**Figure 5:**
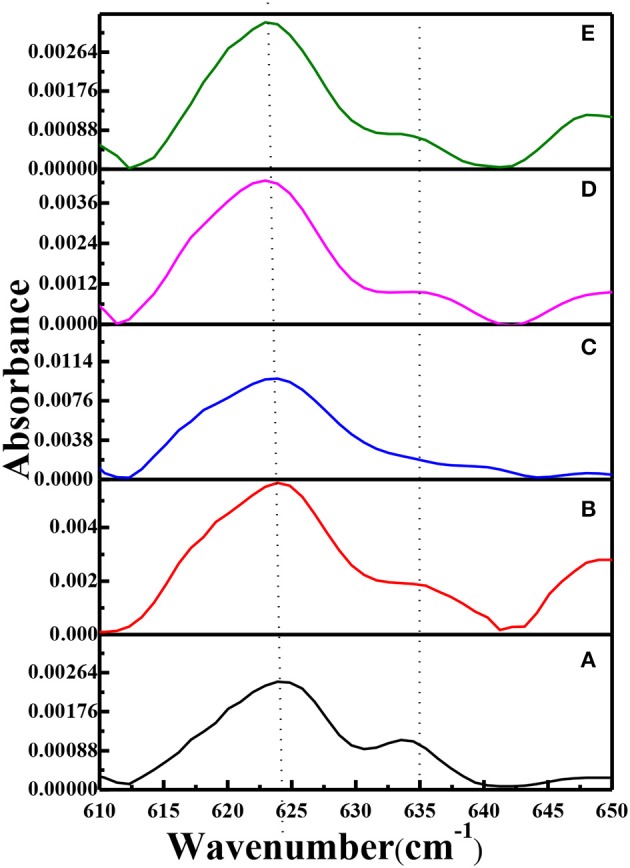
FTIR-ATR spectra of solid composite polymer electrolytes prepared with thermal annealing at 120°C [(a) PEO-LiClO_4_; (b) PEO-LiClO_4_-5% g-C_3_N_4_; (c) PEO-LiClO_4_-10% g-C_3_N_4_; (d) PEO-LiClO_4_-15% g-C_3_N_4_; (e) PEO-LiClO_4_-20% g-C_3_N_4_].

FTIR spectra of PEO, pure g-C_3_N_4_, the PEO-LiClO_4_ solid polymer electrolyte and the PEO-LiClO_4_-g-C_3_N_4_ solid composite polymer electrolyte were shown in Figure S4. And the typical band of LiClO_4_ was identified at 624 cm^−1^. In order to study the effect of g-C_3_N_4_ on the dissociation of LiClO_4_ in the solid composite polymer electrolytes, FTIR-ATR spectra in the range of 610–650 cm^−1^ were carefully analyzed. It was reported that the peaks at ~624 and ~635 cm^−1^ were assigned to the dissociated free anion and the bonded ion pair in LiClO_4_, respectively, Lin et al. (2016). As shown in Figure 5, the absorbance peak of dissociated free anion shifted from 624 to 623 cm^−1^, as the weight percent of g-C_3_N_4_ increased to 15%. It demonstrated that the vibration energy level transition of free ${\text{ClO}}_{4}^{-}$ was easier, due to the interaction of g-C_3_N_4_ and LiClO_4_. It is because the residual –NH_2_ and –NH could coordinate with the lithium ion, which would accelerate the dissociation of LiClO_4_. Moreover, the relative absorbance of peaks at ~624 and ~635 cm^−1^ (labeled as A_624_/A_635_) was obtained from curves in Figure 5. And A_624_/A_635_ was 2.24, 3.01, 4.79, 4.34, and 4.41, respectively, with the weight percent of g-C_3_N_4_ increasing from 0 to 20%. It demonstrated that the introduction of g-C_3_N_4_ could enhance the dissociation of LiClO_4_, with 10% as the optimum content. Also the residual –NH_2_ and –NH groups in g-C_3_N_4_ could form hydrogen bonds with PEO, which would limit the ordered folding of PEO chains and helped to enhance the chain segment motion. As a result, the dissociation of LiClO_4_ was accelerated, due to the coordination between Li^+^ and the ether oxygen. The above factors contributed to the separation of Li^+^ and ${\text{ClO}}_{4}^{-}$ in the solid composite polymer electrolyte. And the dissociation of LiClO_4_ in the solid composite polymer electrolyte is critical for the good ionic conductivity.

### Ionic Conductivity and the Electrochemical Properties of the Solid Composite Polymer Electrolytes

**Figure 6:**
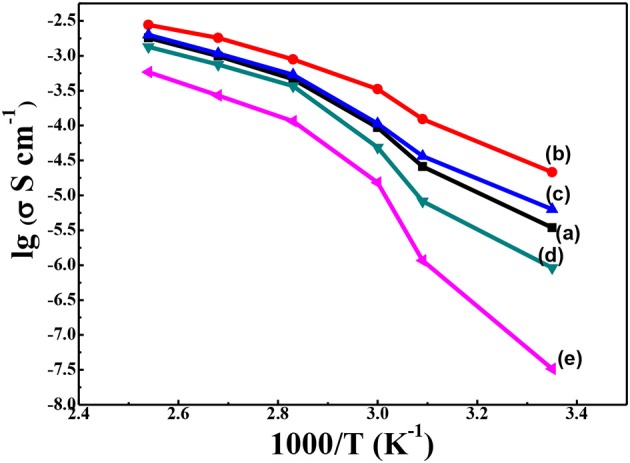
Temperature-dependent ionic conductivity for the solid composite polymer electrolytes with different weight percent of g-C_3_N_4_ prepared with thermal annealing at 120°C [**(a)** 5%; **(b)** 10%; **(c)** 15%; and **(d)** 20%] and the PEO-LiClO_4_ electrolyte **(e)** at the same [EO]: [Li^+^] ratio of 15:1.

In order to confirm our hypothesis of the increasing conductivity by thermal annealing treatment and the addition of g-C_3_N_4_, the ionic conductivity and electrochemistry properties of the solid composite polymer electrolytes were further studied. The ionic conductivity of the solid composite polymer electrolytes with different content of g-C_3_N_4_ was investigated by EIS. The Arrhenius plot of ionic conductivity with temperature from 25 to 120°C was displayed in Figure 6. Compared with that of PEO-LiClO_4_ polymer electrolyte (Figure 6e), significant improvement of ionic conductivity was observed in the annealed solid composite polymer electrolyte. Moreover, the ionic conductivity was firstly increased and then decreased at each temperature with the content of g-C_3_N_4_ increasing from 5 to 20%, as shown in Figures 6a–d. The solid composite polymer electrolyte with 10% g-C_3_N_4_ exhibited the maximum ionic conductivity (1.76 × 10^−5^ S cm^−1^) at 25°C, and the conductivity reached to about 1.08 × 10^−3^ S cm^−1^ at 100°C. And this ionic conductivity at room temperature was much higher than that of the recently reported PEO/g-C_3_N_4_/LiTFSI solid electrolytes (2.3 × 10^−6^ S cm^−1^, at 30°C) (Sun et al., 2019). Also this ionic conductivity is nearly close to that (2.2 × 10^−5^ S cm^−1^ at 28°C) of the PEO based solid composite electrolyte, which needed LiTFSI as the lithium salt and 2D MXene as the filler (Pan et al., 2019). DSC and XRD results demonstrated that the crystallinity of PEO in the solid composite polymer electrolytes was greatly affected by the content of g-C_3_N_4_. And when the content of g-C_3_N_4_ was 10%, PEO gave the minimum crystallinity degree, which was preferred for the high ionic conductivity. Besides effects on the crystallinity of PEO, the residual –NH_2_ or –NH groups exited on the 2D structure of g-C_3_N_4_ could coordinate with Li^+^. Also the porous structure might provide a potential transport path for Li^+^. When the content of g-C_3_N_4_ was 10%, continuous percolating pathways for Li^+^ diffusion might be formed. Nonetheless, when the content of g-C_3_N_4_ was over 10%, the redundant inactive fillers would hinder lithium ionic conduction according to effective medium theory (EMT) (Li W. et al., 2018). Moreover, the modification of PEO-LiClO_4_ using inorganic nanoparticles fillers were always enslaved to the aggregation of particles, which was also harmful for the ionic conducting (Bae et al., 2018a), Evans et al. (1987). As a result, it demonstrated that the thermal annealed PEO-LiClO_4_-g-C_3_N_4_ solid composite polymer electrolyte using 10% g-C_3_N_4_ would give an easy way to achieve excellent ionic conductivity in the solid polymer electrolyte.

**Figure 7:**
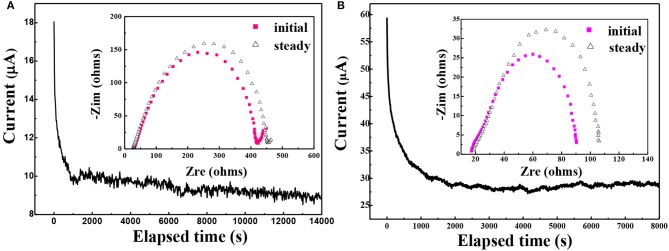
Chronoamperometry of the Li/ PEO-LiClO_4_/Li **(A)** and Li/ PEO-LiClO_4_-10% g-C_3_N_4_/Li **(B)** cells at a potential of 10 mV at 80°C. Inset: the AC impedance spectra of the same battery before and after the polarization.

Since the PEO-LiClO_4_-10% g-C_3_N_4_ gave the best ionic conductivity, it was chosen for the following electrochemical studies. The lithium ion transportation in the solid composite polymer electrolytes was studied as well. And the lithium ion transference number, t_+_, was analyzed according to the method provided by Bruce and coworkers (Evans et al., 1987). Figure 7 showed the relation between time and current crossing a symmetric Li/PEO-LiClO_4_/Li battery (A) and Li/PEO-LiClO_4_-10% g-C_3_N_4_/Li (B) battery polarized by a small voltage of 10 mV at 80°C. The inset was the AC impedance spectra of the same battery before and after the polarization. And the t_+_ can be calculated by the equation: $t_{+}=\frac{I^{s}\left(\text{Δ}V-I^{0}R^{0}\right)}{I^{0}\left(\text{Δ}V-I^{s}R^{s}\right)}\;$, where the *I*^0^ and *I*^*s*^ are the initial current and the steady-state current, respectively; Δ*V* is the potential applied across the cell, and *R*^0^ and *R*^*s*^ are the initial and steady-state interfacial resistances, respectively. Therefore, the t_+_ of the PEO-LiClO_4_ electrolyte was 0.241, while the t_+_ of the annealed PEO-LiClO_4_-g-C_3_N_4_ electrolyte was 0.37. It indicated that the thermal annealing treatment coupled with the addition of g-C_3_N_4_ would greatly enhance the mobility of Li^+^. And this result was in good accordance with the FTIR-ATR results.

**Figure 8:**
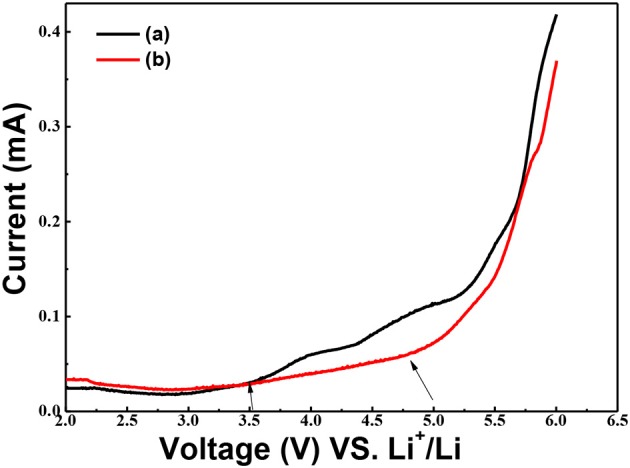
Linear sweep voltammograms of the SS/ PEO-LiClO_4_/Li (a) and SS/ PEO-LiClO_4_-10% g-C_3_N_4_/Li (b) cells at 80°C. The electrolytes were swept in the potential range of 2.0~6.0 V (*VS*. Li^+^/Li) and the scan rate was set as10 mV S^−1^.

Effects of g-C_3_N_4_ on the electrochemical stability of the solid composite polymer electrolytes were characterized by the electrochemical window analysis. The electrochemical stability window of PEO-LiClO_4_ electrolyte and annealed PEO-LiClO_4_-g-C_3_N_4_ electrolyte at 80°C were measured by LSV, as shown in Figure 8. Detected current of PEO-LiClO_4_ electrolyte increased sharply as the applying voltage exceeded 3.5 V, as seen from Figure 8a. But Figure 8b showed that the detected current of PEO-LiClO_4_-g-C_3_N_4_ electrolyte was more stable and displayed a decomposition voltage at about 4.8 V. It implied that the annealed solid composite polymer electrolyte might guarantee a higher working voltage of electrode. And due to the interaction between g-C_3_N_4_ and PEO, the stability of the solid polymer electrolyte was greatly enhanced. It was reasonable to modify the properties of PEO electrolyte through physically thermal annealing treatment and using g-C_3_N_4_ as the filler.

**Figure 9:**
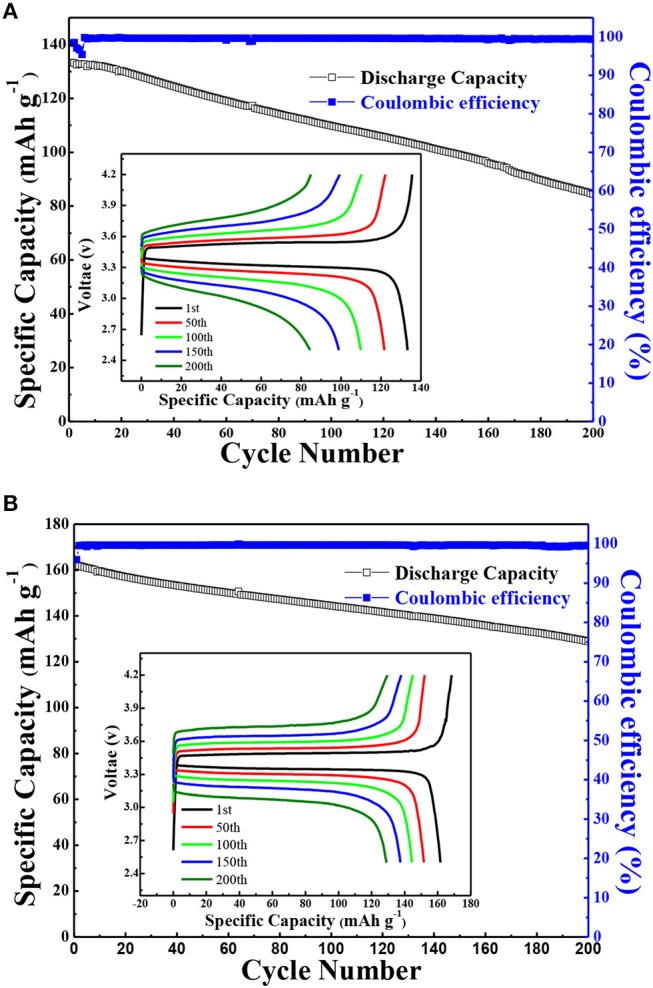
The cycling performance and coulombic efficiencies of LiFePO_4_/Li batteries assembled using PEO/LiClO_4_ electrolyte **(A)** and using the annealed state polymer electrolyte with 10% g-C_3_N_4_ **(B)** at 80°C with a current density of 1 C. And the insert in **(A)** and **(B)** are typical potential VS. specific capacity profiles of the corresponding sample during the cycling performance.

The battery performance of all-solid-state lithium battery (LiFePO_4_/Li) based on the solid polymer electrolyte was also performed. Figure 9 showed the long-term cycling performance and coulombic efficiencies of LiFePO_4_/Li batteries assembled using the annealed state polymer electrolyte with 10% g-C_3_N_4_ and PEO/LiClO_4_ with a current density of 1 C at 80°C. As shown in Figure 9A, the cell based on PEO-LiClO_4_ electrolyte gave an initial discharge capacity of only 133.3 mAh g^−1^, and displayed a capacity retention ratio of 80% after 115 cycles. On the other hand, Figure 9B demonstrated that the cell based on the solid composite polymer electrolyte with a capacity retention ratio of 80% after 200 cycles possessed an initial discharge capacity of 161.2 mAh g^−1^, which was very close to the theoretical capacity of LiFePO_4_ (170 mAh g^−1^). Moreover, this cycling performance was better than the reported LiFePO_4_/Li solid-state batteries based on PEO-LiTFSI-g-C_3_N_4_ polymer electrolytes, which gave an initial discharge specific capacity of 161.3 mAh g^−1^ but cycled for 120 cycles at 60°C (Sun et al., 2019). The coulombic efficiency of batteries based on the solid composite polymer electrolyte during charge-discharge cycling test was as high as 99.7% in Figure 9B, which was higher than that of the battery based on the PEO-LiClO_4_ electrolyte shown in Figure 9A. Moreover, the prevailing experiments using LiFePO_4_ cathode in SPE based solid state lithium batteries were cycled within 3.8 V, in order to guarantee the cycle stability (Nie et al., 2018). In this article, the LiFePO_4_/Li solid battery could operate in the range of 4.2~2.5 V and gave excellent cycle stability. Furthermore, the charge-discharge voltage profiles of the corresponding LiFePO_4_/Li solid battery during the cycling performance were displayed as the insert in Figures 9A,B. The voltage plateau at around 3.48 V was steady in Figure 9B, while a sloping voltage plateau was observed in Figure 9A. And the attenuation of specific capacity during charge-discharge cycling process was more clearly in the LiFePO_4_/Li batteries with PEO/LiClO_4_ electrolyte shown in Figure 9A, compared with that in the LiFePO_4_/Li batteries with annealed PEO-LiClO_4_-g-C_3_N_4_ electrolyte shown in Figure 9B. It implied that the addition of g-C_3_N_4_ enhanced the electrochemical stability of PEO-based solid polymer electrolytes.

**Figure 10:**
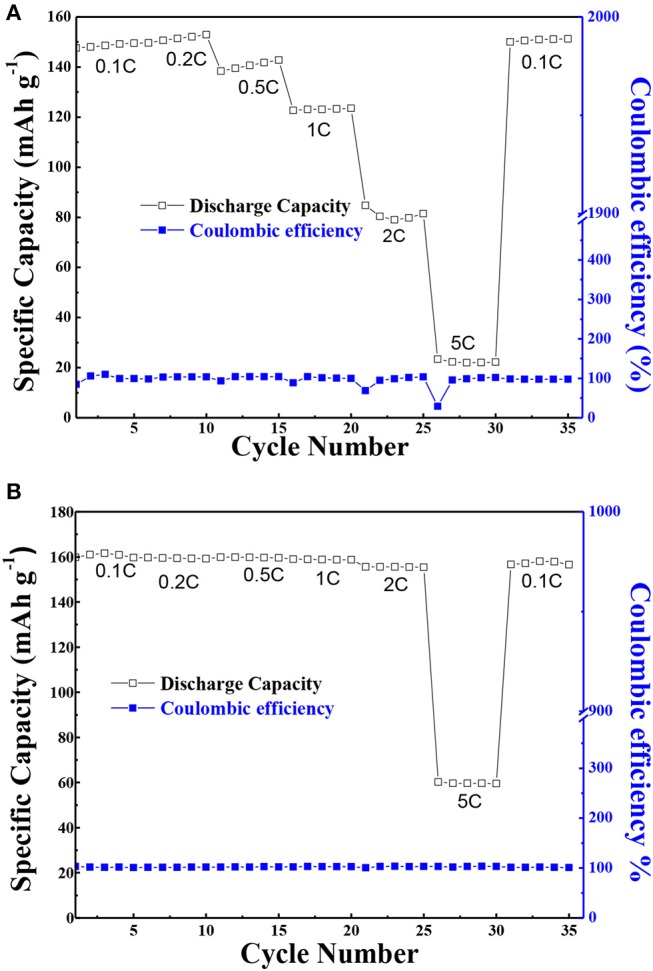
The C-rate performance of LiFePO_4_/Li coin cells at 80°C assembled using PEO/LiClO_4_ electrolyte **(A)** and using annealed state polymer electrolyte with 10% g-C_3_N_4_ **(B)**. And every different C-rate was performed for 5 cycles with a charge current density of 0.1 C.

Moreover, in order to ascertain the usefulness of the solid composite polymer electrolyte, the C-rate cycling property of PEO-LiClO_4_ electrolyte and the annealed PEO-LiClO_4_-g-C_3_N_4_ electrolyte at 80°C were characterized and results were shown in Figure 10. As shown in Figure 10A, the discharge specific capacity of the all solid-state battery based on the PEO-LiClO_4_ electrolyte was 148.0, 150.6, 139.5, 123.1, 80.4, and 22.2 mAh g^−1^, respectively, with the C-rate increasing from 0.1, 0.2, 0.5C, 1, 2 to 5C. And it was obvious that the discharge specific capacity was gradually decreased at higher C-rates, which was reported as a typical characteristic of LiFePO_4_ material (Brutti et al., 2012). The low discharge specific capacity at 5C-rate was attributed to the electrode polarization and solid electrolyte interface caused by the low electronic conductivity, limited diffusion of Li^+^ into the LiFePO_4_ structure (Kumar et al., 2014). On the other hand, Figure 10B showed the performance of the battery with PEO-LiClO_4_-g-C_3_N_4_ electrolyte. It delivered an initial discharge specific capacity of 161.0 mAh g^−1^ at 0.1C-rate. And the discharge specific capacity was 159.5, 159.7, 158.8, and 155.6 mAh g^−1^, respectively, at 0.2C-rate, 0.5C-rate, 1C-rate, and 2C-rate without any obvious attenuation. The battery was even able to deliver a specific capacity of 59.7 mAh g^−1^ even at 5C-rate, which was 168% higher than that of the battery based on the PEO-LiClO_4_ solid polymer electrolyte. Moreover, when the rate returned to 0.1C, the average discharge capacity of the battery was still as high as 157.2 mAh g^−1^. Also the coulombic efficiencies were close to 100%. This result implied that the PEO-LiClO_4_-g-C_3_N_4_ solid polymer electrolyte could facilitate the cycling performance of LiFePO_4_ cathode, due to its good compatibility with the cathode and good lithium ionic transport capability. Moreover, the solid state battery based on the solid composite polymer electrolyte was able to light up several LED lamps under a normal condition as shown in Figure S5. It demonstrated that the annealed PEO-LiClO_4_-g-C_3_N_4_ electrolyte could be an outstanding candidate used in all solid-state lithium batteries.

## Conclusions

To conclude, we reported a novel method to prepare the solid polymer electrolyte with high performance using facile thermal annealing treatment and by addition g-C_3_N_4_ as the modification filler. XRD and DSC results demonstrated that the crystallinity degree of PEO was greatly decreased by the thermal annealing treatment and by interaction between PEO chains and the g-C_3_N_4_ filler. Moreover, the dissociation of LiClO_4_ was obviously enhanced as proved by the FTIR-ATR results. The final morphology of the obtained solid composite polymer electrolyte was even changed with the content of g-C_3_N_4_ increasing and changed by the thermal annealing treatment. When the weight percent of g-C_3_N_4_ was 10% in the annealed solid composite polymer electrolyte, spherulites morphology of PEO crystals was totally destroyed, which would facilitate the compatibility between the solid electrolyte and electrodes. And the crystallinity of PEO was only about 8%, which was helpful for the lithium ion transport in the solid composite polymer electrolyte.

As a result, the obtained solid composite polymer electrolyte with 10% g-C_3_N_4_ achieved an ionic conductivity as high as 1.76 × 10^−5^ S cm^−1^ at 25°C, due to the above microstructure regulation. The electrochemical stable window was increased to about 4.8 V. The lithium ion transference number, t_+_, was changed from 0.24 to 0.37, and increased about 54.8%. Finally, the annealed PEO-LiClO_4_-10% g-C_3_N_4_ electrolyte was also assembled into an all solid-state battery using LiFePO_4_ as the cathode and lithium metal as the anode. And the battery demonstrated excellent performance, with a high initial discharge capacity of 161.2 mAh·g^−1^ and superior cycle stability with a capacity retention ratio of 81% after 200 cycles at 1C at 80°C. Furthermore, the C-rate cycling results also demonstrated that the stability of the assembled solid state battery was markedly improved by using PEO-LiClO_4_-10% g-C_3_N_4_ electrolyte. The above results indicates that the thermal annealing treatment and g-C_3_N_4_ as a novel structure modifier provide an useful and facile way to prepare the state polymer electrolytes used in the all solid-state lithium battery.

## Data Availability

The raw data supporting the conclusions of this manuscript will be made available by the authors, without undue reservation, to any qualified researcher.

## Author Contributions

LS and JY developed the concept and designed the experiment. They contributed equally to this work. XW and JY conducted the experiments. KX, GZ, and AM co-supervised the experiments. JY and LS wrote the manuscript. KX, CS, and WC helped to revise the work critically. All authors listed have made a substantial, crucial and direct contribution to the work, and approved it for publication.

### Conflict of Interest Statement

LS was employed by the company Shaanxi Coal Chemical Industry Technology Research Institute Co. Ltd. The remaining authors declare that the research was conducted in the absence of any commercial or financial relationships that could be construed as a potential conflict of interest.
