# Supplementary material for: High-Performance Solid Composite Polymer Electrolyte for all Solid-State Lithium Battery Through Facile Microstructure Regulation
Source: Front Chem. 2019 May 31;7:388. doi: 10.3389/fchem.2019.00388 (PMC6554539; doi:10.3389/fchem.2019.00388)
Supplement: Supplementary file 1 [file Table_1.docx]

Supporting Information

**High-performance solid composite polymer electrolyte for all solid-sate lithium battery through facile microstructure regulation**

Jingjing Yang^1*#^, Xun Wang^1^, Gai Zhang^1^, Aijie Ma^1^ *and* Weixing Chen^1^

^1^School of Materials and Chemical Engineering, Xi’an Technological University, Xi’an, China.

Le Shao*^2*#^* , Chao Shen^3^ *and* Keyu Xie^3^*^*^*

^2^Shaanxi Coal Chemical Industry Technology Research and Institute Co. Ltd., Xi’an, China.

^3^State Key Laboratory of Solidiﬁcation Processing, Center for Nano Energy Materials, School of Materials Science and Engineering, Northwestern Polytechnical University and Shaanxi Joint Laboratory of Graphene (NPU), Xi’an, China.

*** Correspondence:**

Dr. Jingjing Yang, Dr. Le Shao and Prof. Keyu Xie

yangjingjing@xatu.edu.cn, shaole@sxccti.com, kyxie@nwpu.edu.cn

The morphology of as-prepared g-C_3_N_4_ was characterized by SEM, as shown in Figure S1. The Field emission scanning electron microscopy (FESEM) measurements were carried out with Nova NanoSEM 450. Figure S1 showed that the interpenetrating porous microstructure was formed by g-C_3_N_4_ nanosheets. The diameter of pores was changed from several hundred nanometers to several micrometers.





**Figure S1** SEM image of the prepared g-C_3_N_4_ sample.

The crystalline structure of pure g-C_3_N_4_ was also analyzed by XRD. And the XRD pattern was collected by a XRD-6000 (Shimazu, Japan) X-Ray diffractometer equipped with Cu Kα radiation in the range of 5~50° at a scanning rate of 4°/min. As shown in Figure S2, typical diffraction peak could be observed at 13.1° and 27.5°, respectively. The diffraction peak at 13.1° was ascribed to the in-planar (100) crystalline plane of g-C_3_N_4_. And the diffraction peak at 27.5° was ascribed to the (002) crystal plane, which indicated the layered stacking structure gave a period of 0.32 nm. And this result was in good agreement with the reported g-C_3_N_4_ (Liu et al., 2011).

**Figure S2** XRD pattern of the prepared g-C_3_N_4_ sample.

The thermal stability of as-prepared g-C_3_N_4_ was characterized by TGA, as shown in Figure S3. The onset thermal decomposition temperature was about 446.0^o^C. It implied that the as-prepared g-C_3_N_4_ possessed excellent thermal stability.

**Figure S3** TGA curve of the prepared g-C_3_N_4_ sample in N_2_ atmosphere, with a heating rate of 10^o^C/min.

FTIR spectra of PEO, pure g-C_3_N_4_, the PEO-LiClO_4_ solid polymer electrolyte and the PEO-LiClO_4_-g-C_3_N_4_ solid composite polymer electrolyte were shown in Figure S4. FTIR spectra were obtained on a Bruker Vertex 70 FTIR spectrometer equipped with a DTGS detector. The resolution was 2 cm^-1^ and the scan was repeated for 32 times. The characteristic bands of PEO are indentified at 2892, 1968, 1061, 950 and 840 cm^-1^, as shown in Figure 1(a). The broad weak band around 3235.9 cm^-1^ shown in Figure 1(b) is the N-H stretching bands and the hydrogen-bonding interactions in g-C_3_N_4_. Also the bands at 1637, 1570, 1462 and 1412 cm^-1^ shown in Figure 1(b) are the typical stretching vibration modes of heptazine-derived repeating units in g-C_3_N_4_ (Liu et al., 2011). As shown in Figure 1(c) and (d), the typical band of LiClO_4_ was identified at 624 cm^-1^. And the relative bands intensity in the 1500~800 cm^-1^ reflected the interaction between PEO and g-C_3_N_4_.

**Figure S4**. FTIR spectra of PEO(a), pure g-C_3_N_4_(b), the PEO-LiClO_4_ solid polymer electrolyte (c) and the PEO-LiClO_4_-10% g-C_3_N_4_ composite polymer electrolyte (d).

The assembled all solid-state LiFePO_4_/Li coin cell (CR2025) using PEO-LiClO_4_-g-C_3_N_4_ was successfully able to light up 10 LED lamps at 25^o^C, as shown in Figure S5. It further demonstrated the practicability and reliability of this kind of all solid-state lithium battery.


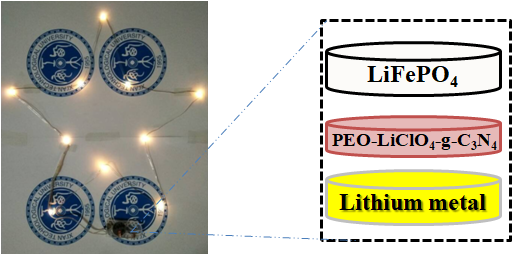


**Figure S5** Illustration of the LiFePO_4_/PEO-LiClO_4_-g-C_3_N_4_/Li all solid-state coin cell (CR2025) for powering 10 LED lamp. Temperature: 25^o^C.

Liu, J.; Zhang, T.; Zhichao Wang; Dawson, G.; Chen, W. (2011).Simple pyrolysis of urea into graphitic carbon nitride with recyclable adsorption and photocatalytic activity. *J. Mater. Chem.*, 21: 14398-14401.doi: 10.1039/C1JM12620B
